# Neuroimmune Function and the Consequences of Alcohol Exposure

**DOI:** 10.35946/arcr.v37.2.15

**Published:** 2015

**Authors:** Fulton T. Crews, Dipak K. Sarkar, Liya Qin, Jian Zou, Nadka Boyadjieva, Ryan P. Vetreno

**Affiliations:** Fulton T. Crews, Ph.D. is John Andrews Distinguished Professor of Pharmacology and Psychiatry and director; Liya Qin, Ph.D., is a research associate; Jian Zou, Ph.D., is a research associate; and Ryan P. Vetrano, Ph.D, is a postdoctoral research associate, all at the Bowles Center for Alcohol Studies, University of North Carolina, Chapel Hill, North Carolina. Dipak K. Sarkar, Ph.D., D.Phil., is Board of Governors and distinguished professor in the Department of Animal Sciences and director of the Endocrine Program, and Nadka Boyadjieva, M.D., Ph.D., D.Sci., is a research professor in the Department of Animal Sciences, both at Rutgers University, New Brunswick, New Jersey.

**Keywords:** Alcohol use, abuse, and dependence, alcohol effects and consequences, alcohol exposure, binge drinking, immunity, neuroimmune responses, neuroimmune genes, neurodegeneration, brain, microglia, stress axis, stress responses, oxidative stress, glutamate receptors, Toll-like receptors, cytokines, high-mobility group box 1, nuclear factor-kappa B

## Abstract

Induction of neuroimmune genes by binge drinking increases neuronal excitability and oxidative stress, contributing to the neurobiology of alcohol dependence and causing neurodegeneration. Ethanol exposure activates signaling pathways featuring high-mobility group box 1 and Toll-like receptor 4 (TLR4), resulting in induction of the transcription factor nuclear factor kappa-light-chain-enhancer of activated B cells, which regulates expression of several cytokine genes involved in innate immunity, and its target genes. This leads to persistent neuroimmune responses to ethanol that stimulate TLRs and/or certain glutamate receptors (i.e., N-methyl-d-aspartate receptors). Alcohol also alters stress responses, causing elevation of peripheral cytokines, which further sensitize neuroimmune responses to ethanol. Neuroimmune signaling and glutamate excitotoxicity are linked to alcoholic neurodegeneration. Models of alcohol abuse have identified significant frontal cortical degeneration and loss of hippocampal neurogenesis, consistent with neuroimmune activation pathology contributing to these alcohol-induced, long-lasting changes in the brain. These alcohol-induced long-lasting increases in brain neuroimmune-gene expression also may contribute to the neurobiology of alcohol use disorder.

To a large extent, signaling processes between neurons in the brain are distinct from signaling mechanisms between cells in the immune system and use different signaling molecules. However, some proteins first discovered within the immune system act as both peripheral immune-signaling molecules and brain-signaling molecules. These neuroimmune factors include various cytokines, Toll-like receptors (TLRs), and high-mobility group protein box 1 (HMGB1). In the brain, both neurons and supporting glial cells (both astrocytes and microglia) contribute to the release of and responses to these neuroimmune factors. Neuroimmune signaling in the brain not only is a part of the innate immune response, but its effects also persist for long periods and could contribute to long-lasting changes in neurobiology.

Studies found that brain neuroimmune signaling is activated in models of binge drinking and neurodegeneration, suggesting another pathway through which alcohol may affect brain function. This review defines the roles of various cellular compartments and signaling molecules involved in neuroimmune activation, including the role of the stress axis in the communication between the central and peripheral immune systems and in sensitizing the neuroimmune response to alcohol. The article also will offer evidence from animal studies and postmortem human alcoholic brain studies that neuroimmune signaling may increase alcohol drinking and risky decision making and (in alcohol-treated animals) blunt the ability to change, decreasing behavioral flexibility.

## Neuroimmune Signaling in the Alcoholic Brain

### Monocytes and Innate Immune Genes

**Figure 1:**
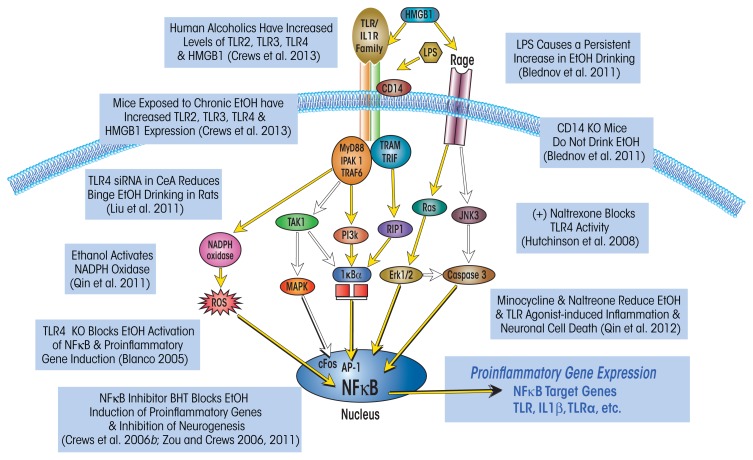
Simplified schematic of the Toll-like receptor (TLR) and the receptor for advanced glycation end products (RAGE) signaling cascades. Stimulation of TLRs with high-mobility group box 1 protein (HMGB1) and other inflammation-inducing agents leads to the generation of reactive oxygen species (ROS) and downstream activation of the transcription factor nuclear factor kappa-light-chain-enhancer of activated B cells (NF)-κB. Similarly, HMGB1 activation of the RAGE receptor results in downstream activation of NF-κB and induction of ROS. Transfer of NF-κB to the nucleus induces proinflammatory gene expression, neuroimmune induction, and cell death. Expression of several TLRs (i.e., TLR2, TLR3, and TLR4) and HMGB1 is upregulated in the postmortem human alcoholic brain and mouse brain following exposure to ethanol (Crews et al. 2013); this is accompanied by an upregulation of NADPH oxidase expression (Qin et al. 2011). Interestingly, blockade of neuroimmune signaling, either genetically (Blanco 2005) or pharmacologically (Crews et al. 2006*b*; Qin et al. 2012; Zou and Crews 2006, 2011), prevents ethanol-induced neuroimmune-gene induction and neurodegeneration. The neuroimmune system also contributes to alcohol-drinking behavior, because activation (Blednov et al. 2001) or blockade of this system (Blednov et al. 2011; Liu et al. 2011) increases and decreases self-administration, respectively. NOTE: AP-1: activator protein-1; CD14: cluster of differentiation 14; ERK: extracellular signal–regulated kinase; IKK: inhibitor of NF-κB; IRAK 1: interleukin-1 receptor–associated kinase 1; JNK: c-jun N-terminal kinases; LPS: lipopolysaccharide; MAPK: mitogen-activated protein kinase; MyD88: myeloid differentiation primary response gene 88; NADPH oxidase: nicotinamide adenine dinucleotide phosphate-oxidase; PI3K: phosphatidylinositol-4,5-bisphosphate 3-kinase; RIP: receptor interacting protein; TAK1: transforming growth factor beta–activated kinase 1; TRAF: tumor necrosis factor receptor–associated factor; TRAM: TRIF-related adaptor molecule; TRIF: TIR-domain-containing adaptor–inducing interferon-beta. SOURCE: Adapted from Crews et al. 2011, 2013.

Innate immune genes are associated with rapid first-line responses to infections that involve primarily immune cells called monocytes (e.g., the acute-phase response). These responses include increases in multiple cytokines as well as in their cellular receptors. Together, these changes amplify expression of a large number of genes through kinase signaling pathways that converge on two transcription factors called nuclear factor kappa-light-chain-enhancer of activated B cells (NF-κB) and activator protein-1 (AP-1). NF-κB and AP-1 promote expression of innate immune cytokines, such as tumor necrosis factor alpha (TNF-α) and interleukin 1 beta (IL-1β), as well as of TLRs and cytokine receptors (see figure 1). In addition, innate immune responses include the activation of proteases and oxidases, particularly cyclooxygenase and nicotinamide adenine dinucleotide phosphate (NADPH) oxidase,^1^ as well as of major histocompatibility complex (MHC) signaling molecules, such as beta-2 microglobulin.

The NF-κB–mediated transcription of proinflammatory genes, in turn, is amplified within and across cells by induction of TLRs and cytokine receptors (e.g., those that belong to the IL-1β receptor family), which induce innate immune gene expression. Amplification of innate immune gene induction across cells and tissues can cause pathology, such as sepsis. Sepsis and systemic inflammatory-response syndrome involve a “cytokine storm.” This potentially fatal innate immune reaction consists of positive feedback loops between cytokines and immune and tissue cells, resulting in highly elevated levels of cytokines in the blood, multiorgan failure, and death (Osterholm 2005). Models of sepsis that involve activation of an acute phase-like response lead to increases in the levels of multiple cytokines in the blood that occur in two distinct phases. First, both TNF-α and IL-1β levels increase during the first several hours after infection but then subside. Subsequently, levels of HMGB1, a ubiquitously expressed, cytokine-like protein that can activate TLR4 and potentiate cytokine responses, increase about 16 hours after infection and remain elevated for several days (Wang et al. 2001). In mouse models, sepsis-induced death that occurs several days after infection is associated with HMGB1 and is prevented by treatment with antibodies blocking HMGB1. Survivors of sepsis show prolonged increases in serum HMGB1 and cognitive deficits that can be prevented with HMGB1-antibody treatment (Chavan et al. 2012). About half of the patients released from the hospital after surviving a cytokine storm–sepsis insult die within 5 years (Quartin et al. 1997). Thus, innate immune responses can be long lasting and can induce pathology long after they initially have been activated. However, although most studies support a central role for NF-κB–mediated transcription of proinflammatory cytokines, proteases, and oxidases in the innate immune response, both the precise mechanisms that regulate individual cell or cytokine activation and the contributions of tissues and cells in vivo to amplification of specific innate immune genes are poorly understood.

Monocytes are the primary cells mediating the innate immune response. They are found in all tissues, including the brain. Monocytes in the brain, which also are referred to as microglia, fall into two main categories: proinflammatory M1 monocytes/microglia and trophic M2 monocytes/microglia. M1 monocytes/microglia participate in the acute proinflammatory responses of the innate immune system; in addition, they also convey signals to the adaptive immune cells (i.e., T and B cells) through the MHC molecules they carry on their cell surface. These signals help create a persistent sensitization to pathogens (e.g., in the form of antibodies that mediate immunization). These proinflammatory effects occur in response to pathogens as well as tissue damage, cell death, and degeneration. Thus, M1 microglia and other monocyte-like cells consistently express multiple cytokine receptors and TLRs that, when activated, induce innate immune genes, such as proinflammatory cytokines, proteases, and oxidases, which help to break down, process, and remove damaged cells and tissue. In contrast, the M2 monocytes/microglia mediate a delayed response that initiates wound-healing trophic signaling and seem to be critical for healing. Both monocytes in general and brain microglia in particular can have both proinflammatory M1 and trophic M2 phenotypes (Colton 2009; Michelucci et al. 2009).

Although the M1 and M2 phenotypes are poorly understood, monocyte proinflammatory activation clearly is linked to NF-κB–mediated transcription of multiple innate immune genes. Activation of monocyte NF-κB by both pathogens and tissue damage involves TLR4 (see figure 1). This receptor responds to endotoxin released by certain bacteria (e.g., lipopolysaccharide [LPS]) as well as to HMGB1. Proinflammatory gene induction also is amplified by cytokine– receptor-activated release of HMGB1 that further contributes to innate immune gene induction.

The role of these innate immune-signaling molecules is well characterized within the immune system, but only recently these molecules have been discovered to also contribute to brain signaling. Thus, studies have indicated that MHC molecules contribute to brain development (Huh et al. 2000), to most neuro-degenerative diseases (Gage 2002; Glass et al. 2010), and to alcohol and other drug dependence (Crews 2012). Neuroimmune signaling in the brain has not been extensively studied, and most knowledge on this subject is based on the assumption that monocyte responses elsewhere in the body reflect microglial and brain innate immune responses.

### The Immune Response in the Brain

The immune system is not normally active in the healthy brain. Thus, the healthy normal brain does not contain antibodies and has only one type of immune cell, the microglia. During fetal development, neurons, astrocytes, and all other brain cells are formed from one embryonic structure (i.e., the ectoderm), whereas microglia migrate from another embryonic structure (i.e., the mesoderm) to the brain at a specific time (Ginhoux et al. 2010). In the healthy brain, the number of ramified or “resting” microglia equals that of neurons, and these cells contribute to the integration of sensory systems and overall survey of the brain milieu (Raivich 2005). Along with astrocytes, they modulate important metabolic, trophic, and synaptic functions in addition to responding to brain–damage-induced neuroimmune responses (Farina et al. 2007; Streit et al. 2004). Microglia respond to endogenous or exogenous insults with distinct morphological changes in shape (i.e., they develop “bushy” or “amoeboid-like” phenotypes) as well as with marked alterations in gene expression, including proinflammatory innate immune-response genes (Graeber 2010). However, it sometimes is unclear whether microglia are responding to a brain insult or causing it through the release of proinflammatory cytokines. Microglia respond to and signal through both neuroimmune and neurotransmitter signals. For example, acetylcholine—an important neurotransmitter involved in multiple brain functions, including cognition—inhibits proinflammatory activation in both peripheral monocytes and brain microglia and has anti-inflammatory effects.

**Figure 2:**
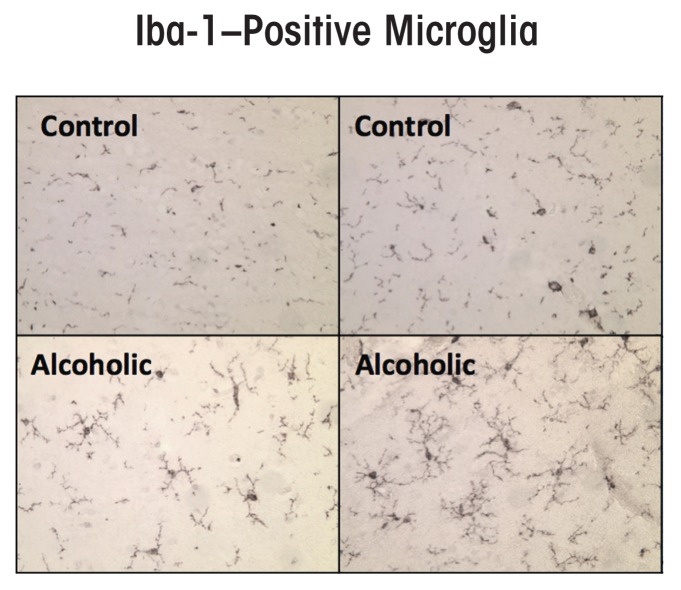
Microglial activation, as indicated by expression of the microglial marker Iba-1, is increased in postmortem alcoholic brain. The photomicrographs depict microglia from postmortem brain samples of alcoholics and control subjects. The number of Iba-1–positive microglia (dark stains) is higher in the alcoholic than in the control samples. SOURCE: He and Crews 2008.

Some studies found an increase in the expression of the microglial marker Iba-1 in the brains of alcoholic individuals (see figure 2) (He and Crews 2008), suggesting that microglia contribute to the neurobiology of alcoholism. Microglia in postmortem human alcoholic brain and chronic alcohol-treated mouse and rat brain show increased MHC gene expression, but not the bushy or phagocytic activation profiles associated with marked brain damage. Chronic ethanol treatment also increases microglial TLR4 expression (Vetreno et al. 2013). Thus, microglia are the only immune cells in healthy brain and are integrated into the brain’s responses to both neurotransmitters and neuroimmune signals. They also seem to contribute to chronic alcohol-induced responses.

## Alcohol, Neuroimmune Signaling, and Neurodegeneration

Chronic binge-drinking models repeatedly found that ethanol exposure increases the expression of a variety of neuroimmune genes in the brain and that these alterations may persist over long periods (see figure 1). For example, one study found that chronic ethanol exposure induced the neuroimmune gene cyclooxygenase 2 (COX2) in multiple cortical and limbic brain regions long after physical signs of withdrawal had subsided (Knapp and Crews 1999). However, ethanol did not induce COX2 in transgenic mice lacking TLR4, suggesting that this process involves TLR4 (Alfonso-Loeches et al. 2010). Chronic alcohol exposure also altered the activity of NF-κB and another regulatory protein, cyclic AMP-responsive element binding protein (CREB). Specifically, ethanol treatment of HEC brain slice cultures increased NF-κB binding to DNA probes modeling gene promoter regions and decreased CREB binding to DNA probes modeling CREB-responsive gene promoter DNA (Zou and Crews 2006).

The CREB family of transcription factors is activated by phosphorylation; they promote neuronal survival, protecting neurons from excitotoxicity and apoptosis by regulating the transcription of pro-survival factors (Lonze and Ginty 2002; Mantamadiotis et al. 2002). Conversely, NF-κB is known widely for its ubiquitous roles in inflammatory and immune responses (O’Neill and Kaltschmidt 1997). Accordingly, NF-κB and CREB have different target genes. For example, CREB targets the neuropeptide Y and brain-derived neurotrophic factor (BDNF) genes, both of which are involved in promoting neuronal growth and resilience to insults, including protection against excitotoxicity and neuronal death (Lonze and Ginty 2002). Regular excitation of neurons increases synaptic plasticity related to CREB and induces synaptic proteins and BDNF. In contrast, excessive excitation triggers activation of certain extrasynaptic receptors for the neurotransmitter glutamate (i.e., *N*-methyl-d-aspartate [NMDA] receptors) and excitotoxicity, resulting in either rapid or delayed neuronal death, which is associated with reduced CREB (Hardingham and Bading 2010). Chronic ethanol exposure interferes with the normal functions of CREB. Thus, the levels of CREB phosphorylation and CREB–DNA binding as well as of the target gene BDNF all were decreased in the rat frontal cortex following a 24-hour withdrawal from chronic ethanol exposure (Pandey et al. 1999, 2001). In addition, neuropeptide Y levels were reduced in the cortex following ethanol treatment, an effect that was accompanied by reduced levels of phosphorylated CREB (Bison and Crews 2003).

The reciprocal relationship between NF-κB and CREB transcription sensitizes neurons to excitotoxicity (Zou and Crews 2006). This reciprocal relationship appears to result from the actions of kinases, such as protein kinase A, which activate CREB transcription but inhibit NF-κB activation. However, the reciprocal relationship also may represent differences between neuronal and glial signaling pathways, because regulation of CREB transcription principally occurs in neurons whereas NF-κB activation of pro-inflammatory genes primarily occurs in microglia.

In summary, ethanol can directly increase NF-κB–mediated transcription of proinflammatory genes in the brain as well as decrease trophic protective-factor transcription by reducing CREB transcription. Together, these effects decrease the brain’s resilience to insults.

### Roles of HMGB1 and TLR4

Ethanol induces neuroimmune genes through multiple mechanisms. One mechanism involves alcohol-induced release of HMGB1,^2^ which increases NF-κB–mediated transcription of proinflammatory cytokines (Crews et al. 2013; Zou and Crews 2014). Several transmitters and neuroimmune-signaling receptors as well as neuronal excitability increase the release of HMGB1 (Maroso et al. 2010). This protein is a TLR4 agonist that acts through multiple signaling mechanisms in the brain, thereby influencing astrocytes and microglia, as well as neurogenesis, neurite growth, and excitability in adjacent neurons. HMGB1 released by neuronal activity stimulates TLR4 receptors, resulting in IL-1β release and increased phosphorylation of a subunit of the NMDA receptor (i.e., the NR2B subunit), which in turn increases susceptibility to seizures (Maroso et al. 2010; Vezzani et al. 2011). Actively released HMGB1 is acetylated, and ethanol increases HMGB1 acetylation in brain slice cultures. The acetyl-HMGB1 initially is found primarily in the cell’s cytosol, likely in vesicles, before its concentration in the surrounding fluid increases progressively, consistent with neuronal release (Zou and Crews 2014). The importance of ethanol-induced release of HMGB1 and resulting TLR4 activation to ethanol-induced neurodegeneration and behavioral pathology was demonstrated in studies using cells and animals that no longer produced TLR4 (i.e., TLR4 knockout cells and mice). The experiments showed that knockout of TLR4 markedly blunted chronic–ethanol-induced neurodegeneration and induction of proinflammatory gene expression (Alfonso-Loeches et al. 2010; Blanco et al. 2005; Fernandez-Lizarbe et al. 2009; Pascual et al. 2011; Valles et al. 2004).

Additional studies found that ethanol treatment induced neuroimmune genes in microglia and astrocyte primary cultures as well as in mice and that this induction was dependent on the expression of TLR4. These receptors are always present on microglia, making microglia a key component of drug-induced neuroimmune activation (Alfonso-Loeches and Guerri 2011; Schwarz and Bilbo 2013). In addition, TLR4 is integral to ethanol- nduced dopamine release (Alfonso-Loeches and Guerri 2011), damage to white matter (Alfonso-Loeches et al. 2012), and other pathologies associated with chronic–ethanol-induced changes in the brain (Pascual et al. 2011). In cultured cells, ethanol treatment increases innate immune gene expression in a time-dependent fashion, mimicking responses to LPS or IL-1β administration, although ethanol induces a much smaller response (Crews et al. 2013). In vivo, ethanol induces neuroimmune genes in the brains of wild-type mice, but not TLR4 knockout mice (Alfonso-Loeches et al. 2010). These studies support the hypothesis that TLR4 signaling is critical to many of the effects of alcohol on the brain.

It is not clear why signaling through TLR4 but not via other cytokine receptors seems to contribute significantly to ethanol responses, because all of these receptors generally belong to the same receptor superfamily (i.e., the TLR–IL1-R superfamily) (Wald et al. 2003) and share kinase cascades in monocytes and microglia that all converge upon NF-κB. The findings suggest that the TLR4s on neurons or other brain cells may have some unique properties that differ from NF-κB activation by receptors for TNF-α, IL-1β, and other cytokines (e.g., TNF receptor) which induce NF-κB transcription of proinflammatory cytokines. Further complicating the picture, the TLR4 signaling pathway is not the only one affected by ethanol exposure. Vetreno and colleagues (2013) found that chronic intermittent treatment of adolescent rats also led to persistent increases in the expression of another receptor stimulated by HMGB1, called receptor for advanced glycation end products (RAGE) (see figure 1). Although the mechanisms remain complicated, together these studies suggest that HMGB1–TLR4 and perhaps RAGE signaling (which are found on multiple brain cells types) as well as neuronal–glial neuroimmune signaling and microglial–astrocyte activation all contribute to alcohol-induced brain damage.

### Effects of Acute vs. Chronic Ethanol Exposure

Although chronic alcohol treatment increases proinflammatory gene expression in the brain through activation of TLR4, this is confounded by acute alcohol inhibition of TLR4 signaling in monocytes and possibly other cells. Time-dependent acute and chronic opposing effects of ethanol confound many studies (Crews et al. 2006*a*, 2011; Szabo and Mandrekar 2009). Acute ethanol suppresses the innate immune response to LPS, a TLR4 agonist, in both in vivo and in vitro models. For example, LPS-induced TNF-α and IL-1β production is blunted in blood monocytes obtained from healthy human volunteers after acute alcohol exposure (Crews et al. 2006*a*; Szabo et al. 1993, 1995, 2001). In animal models, acute ethanol exposure attenuates the TNF-α, IL-1β, and IL-6 immune responses to LPS (Pruett et al. 2004). Similarly, in in vitro models, addition of ethanol (25 mM) just before LPS blunts induction of TNF-α (Szabo et al. 1993, 1995, 2001). In contrast, chronic in vitro ethanol exposure of astrocytes, microglia, and brain slices induces NF-κB transduction of proinflammatory genes through activation of TLR4 signaling (Crews et al. 2013; Fernandez-Lizarbe et al. 2009; Zou and Crews 2014).

While it is unclear if the presence of acute ethanol exposure antagonizes TLR4 on all cell types, other TLRs are not acutely blocked by ethanol (Crews et al. 2006*a*). Upregulation of TLRs by chronic alcohol treatment can lead to sensitization. In mice, binge treatment with ethanol for 10 days (5 g/kg/day), followed by LPS 24 hours later when alcohol had cleared, resulted in a marked increase in proinflammatory gene induction (Qin and Crews 2012*b*). Ethanol treatment increased the responses to LPS-induced proinflammatory cytokines in liver, blood, and brain. The responses were transient in blood and liver but were long lasting in brain. Similarly, chronic 10-day alcohol treatment sensitized mice to the proinflammatory response to Poly:IC, a compound that activates TLR3 (Qin and Crews 2012*a*). Thus, the effects of ethanol on brain neuroimmune signaling are in part related to increases in TLRs (see figure 1) that increase neuroimmune signaling and cytokines, such as IL-1β, during chronic ethanol treatment, although the presence of alcohol can blunt TLR4 responses during intoxication.

### Ethanol Induction of HMGB1–TLR Signaling in the Brain

**Figure 3:**
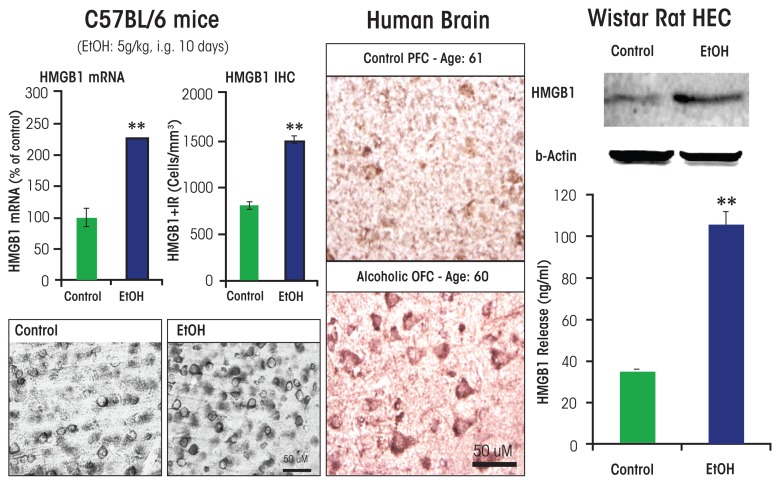
Alcohol increases high-mobility group box 1 (HMGB1) expression in mouse brain, and human brain and induces HMGB1 release from rat brain slices. **(Left)** Chronic ethanol treatment of mice for 10 days increases expression of HMGB1 mRNA and protein. **(Middle)** Postmortem human alcoholic orbitofrontal cortex (OFC) has significantly more HMGB1-immunoreactive cells than seen in age-matched moderately drinking control subjects. **(Right)** Ethanol causes the release of HMGB1 into the media from hippocampal-entorhinal cortex (HEC) slice culture. NOTE: ** P < 0.01, relative to the corresponding control group. SOURCE: Adapted from Crews et al. 2013.

As mentioned previously, studies investigating the mechanisms of ethanol induction of proinflammatory genes in the brain have shown that chronic ethanol increases expression of TLRs as well as the TLR4 receptor agonist HMGB1. Studies of chronic 10-day ethanol treatment of mice (Crews et al. 2013), chronic in vitro treatment of rat brain-slice cultures (Zou and Crews 2014), and analyses of postmortem human alcoholic brain (Crews et al. 2013) all found increased expression of HMGB1, TLR4, TLR3, and TLR2 (see figure 3).^3^ Increases in receptors and agonists are common in innate immune signaling, and these findings suggest that chronic alcohol, through induction of HMGB1 and TLR4 as well as the less well characterized RAGE receptor, may contribute to increases in neuroimmune-gene expression. Brain-slice culture experiments found that ethanol could induce HMGB1 release, which then increased proinflammatory gene expression. This process could be blocked by pharmacological antagonists or knockdown of TLR4 (Crews et al. 2013; Zou and Crews 2014). Studies in adolescent rats (Vetreno and Crews 2012), adolescent mice (Coleman et al. 2014), and adult mice (Qin et al. 2007, 2008, 2013) found long-lasting increases in neuroimmune-gene induction following alcohol treatment.

**Figure 4:**
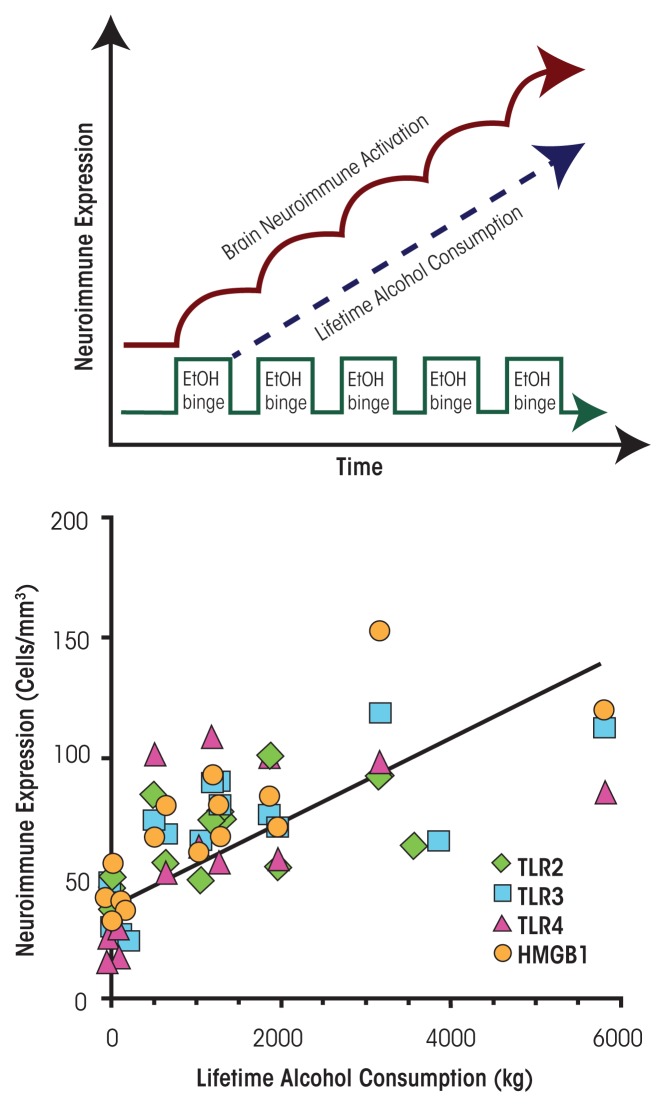
Cycles of chronic alcohol consumption lead to persistently increased neuroimmune-gene expression. **(Top)** Repeated ethanol (EtOH) binges result in increased brain neuroimmune activation (i.e., microglial and astrocytic activation as well as upregulated neuroimmune-gene expression). **(Bottom)** In humans, lifetime alcohol consumption is positively correlated with neuroimmune signal immunoreactivity. Symbols indicate levels of Toll-like receptor (TLR) 2, TLR3, TLR4, and high-mobility group box 1 (HMGB1) in individual moderate drinkers and alcoholics. Results for moderate drinkers are clustered along the Y-axis because of their low lifetime alcohol consumption and similar neuroimmune expression. Alcoholic subjects show a more than 10-fold variation in lifetime alcohol consumption as well as considerable variation in expression of all four neuroimmune genes. NOTE: Correlations are as follows: TLR2: *r* = 0.66 (*p* < 0.01); TLR3: *r* = 0.83 (*P* < 0.001); TLR4: *r* = 0.62 (*P* < 0.01); HMGB1: *r* = 0.83 (*P* < 0.001). SOURCE: Crews et al. 2013.

In humans, levels of HMGB1 and TLR expression in specific brain regions (e.g., the orbitofrontal cortex) have been shown to correlate with lifetime alcohol consumption (Crews et al. 2013) (see figure 4). Alcoholic subjects who vary greatly in the duration and amounts of active drinking bouts exhibited a large variation in lifetime alcohol consumption that correlated with increased HMGB1–TLR expression in the frontal cortex. In contrast, moderate-drinking humans consumed much less alcohol than alcoholics and exhibited much lower HMGB1–TLR expression. This interesting correlation only could occur if ethanol induction of HMGB1– TLR was persistent and cumulative with binge-drinking episodes (see figure 4). Together, these studies suggest that HMGB1–TLR4 signaling is increased by chronic binge drinking, contributing to the persistent and sustained induction of proinflammatory signaling in brain.

## Mechanisms of Neurodegeneration Related to Alcohol’s Effects on Neuroimmune Signaling in the Brain

### Role of NADPH Oxidase and Oxidative Stress

One innate immune gene induced by ethanol and LPS is NADPH oxidase, a multi-subunit enzyme that catalyzes the formation of the reactive oxygen species (ROS), superoxide, and thereby increases oxidative stress. NADPH oxidase first was characterized as a phagocytic oxidase in monocytes, where it was hypothesized to contribute to the oxidation of infectious agents. The superoxide produced by NADPH oxidase can increase NF-κB transcription, thereby creating another amplifying loop of proinflammatory signaling (see figure 1). More recent studies have found that there are multiple genes and forms of NADPH oxidase.

Qin and Crews (2012*b*) discovered that LPS and ethanol can increase expression of NADPH oxidase subunits, particularly the superoxide-forming gp91^phox^ subunit, in the brain and that ethanol treatment of mice increased superoxide formation in the brain as well as neuronal death. Inhibition of oxidases both reduced superoxide formation and protected against alcohol-induced neuronal death. Other studies in mice demonstrated that LPS treatment induced neuroimmune-gene expression, NADPH-oxidase activity, and oxidative stress that persisted for at least 20 months and led to neurodegeneration (Qin et al. 2013). Prolonged induction of NADPH oxidase and oxidative stress in the brain could contribute to the persistent increase in NF-κB transcription observed after alcohol exposure, because ROS can activate NF-κB. These findings are consistent with the hypothesis that oxidative stress, by inducing innate immune genes, significantly contributes to alcoholic brain damage and alcoholic neurodegeneration.

In addition to enhancing ROS levels, alcohol exposure decreases endogenous antioxidant levels, thereby reducing the body’s natural defense against ROS and again increasing oxidative stress (Henderson et al. 1995). Specifically, ethanol decreases the levels of the antioxidant glutathione and the cellular activity of antioxidative enzymes, such as glutathione peroxidase, catalase, and superoxide dismutase. Furthermore, a synthetic superoxide dismutase/catalase mimetic (EUK-134) and a water-soluble analog of vitamin E (Trolox), both of which are well-known antioxidants, protected developing hypothalamic neurons from oxidative stress and cellular apoptosis caused by ethanol-treated microglia medium (Boyadjieva and Sarkar 2013).

### Role of Hyperexcitability and Excitotoxicty

**Figure 5:**
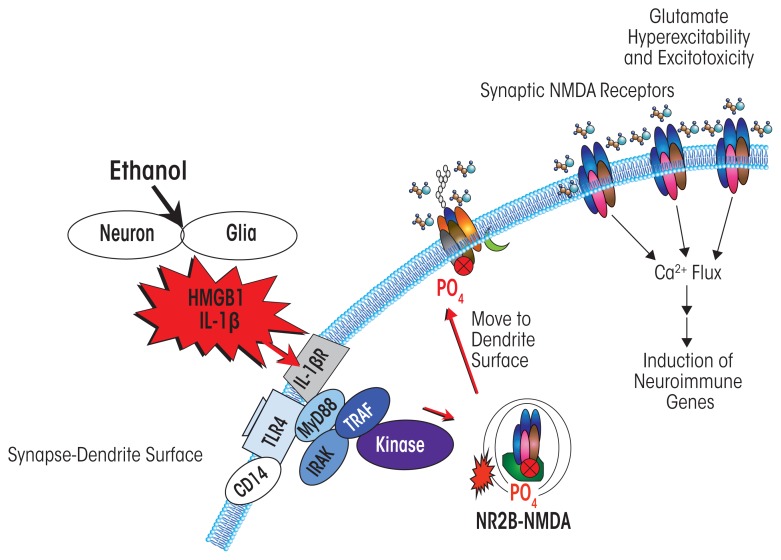
Simplified schematic depicting how neuroimmune signaling leads to neuronal hyperexcitability and the neurobiology of addiction. Alcohol and stress activate neurons and glia in the central nervous system, resulting in the release of various neuroimmune signals (e.g., high-mobility group box 1 [HMGB1] and interleukin-1beta [IL-1β]) that activate neuroimmune receptors (e.g., Toll-like receptors [TLRs]). Neuroimmune receptor stimulation leads to phosphorylation, and thus activation, of glutamatergic *N*-methyl-d-aspartate (NMDA) receptors that are transported to the cell surface (Iori et al. 2013; Maroso et al. 2010). The increased number of NMDA receptors increases Ca^2+^ flux, triggering further induction of neuroimmune genes, and also promotes glutamate hyperexcitability and excitotoxicity.

Another mechanism contributing to alcoholic neurodegeneration and associated with HMGB1–TLR4 signaling is the excessive stimulation of receptors that results in neuron damage and cell death (i.e., excitotoxicity). Chronic ethanol treatment of neurons leads to increased sensitivity to excitotoxicity (Chandler et al. 1994). This effect primarily involves the neuro-transmitter glutamate and its receptors. However, the relationship between ethanol and glutamate receptors is complex. Thus, although ethanol enhances overall glutamate excitotoxicity, in neuronal primary cultures it blocks excitotoxicity associated with a specific type of glutamate receptor (i.e., the NMDA receptor). This is consistent with many studies finding that ethanol inhibits NMDA receptors (Chandler et al. 1998). Yet at the same time, HMGB1–TLR4 signaling (Balosso et al. 2014) and IL-1β receptor signaling (Viviani et al. 2003)—both of which, as described above, are induced by chronic ethanol—increase NMDA receptor-mediated calcium flux, neuronal excitability, and excitotoxicity through activation of kinase signaling cascades, including activation of Src kinase and tyrosine-kinase (see figure 5). Furthermore, Suvarna and colleagues (2005) found that ethanol increases NMDA excitability in the hippocampus through kinase activation that alters receptor trafficking, leading to increased numbers of NMDA receptors containing the NR2B subunit at the synapse.

Another mechanism through which chronic ethanol induces hyperexcitability involves neuroimmune inhibition of glial glutamate transporters (Zou and Crews 2005). Thus, in brain-slice cultures, ethanol potentiates excitotoxicity by causing blockade of the molecules that normally remove glutamate from the synapse into glial cells and may perhaps even induce glutamate release from those cells (Zou and Crews 2006, 2010).

As indicated above, ethanol causes HMGB1 release, creating hyperexcitability that disrupts synaptic plasticity and sensitizes to excitotoxicity. HMGB1 is massively released during brain damage, resulting in persistent neuroimmune-gene induction (Kim et al. 2006). Maroso and colleagues (2010) found that increased HMGB1 release was associated with hippocampal excitability that caused seizures, leading to persistent increases in HMGB1 and excitability. Ethanol has modest cumulative effects with repeated chronic exposure, further exacerbating excitability and excitotoxicity resulting from increased neuroimmune signaling. Thus, the global neurodegeneration associated with alcoholism, with the most severe losses observed in the frontal cortex, is secondary to the persistent and progressive neuroimmune activation.

## Neuroimmune-Gene Expression in Postmortem Human Alcoholic Brain

In addition to the HMGB1–TLR4 signaling cascade, multiple other proinflammatory genes are increased and have been detected postmortem in the brains of alcoholics. Initial human brain studies focused on microglia and the proinflammatory cytokine monocyte chemotactic protein-1 (MCP-1, also known as CCL2), which among the cytokines tested was induced most robustly by ethanol in brain-slice cultures (Zou and Crews 2012). Additional studies also showed increased levels of MCP-1 protein in the ventral-tegmental area, amygdala, nucleus accumbens, and hippocampus (He and Crews 2008). In addition to MCP-1, expression of the microglial marker Iba-1 also was increased. These studies indicate that neuroimmune-gene expression is increased in the human alcoholic brain.

Subsequent studies focusing on the prefrontal cortex, specifically the orbital frontal cortex (OFC), found increased levels of HMGB1 as well as TLRs (specifically TLR2, TLR3, and TLR4) in postmortem alcoholic brain (Crews et al. 2013). Furthermore, NADPH oxidase was increased in alcoholic OFC, consistent with increased oxidative stress as found in mice. The HMGB1 receptor RAGE also was increased in postmortem human alcoholic brain (Vetreno et al. 2013). Finally, studies detected increased IL-1β inflammasome markers in the hippocampus of postmortem alcoholic brains that could contribute to loss of neurogenesis. These observations indicate that multiple neuro-immune genes are increased in alcoholic brain and likely contribute to neurodegeneration and the neurobiology of alcoholism in humans.

Researchers also investigated the relationship between alcohol drinking and neuroimmune-gene expression in alcoholics and control subjects. Interestingly, two forms of correlations were found linking neuroimmune-gene expression to alcohol consumption and alcoholism. The first correlation involved the age at drinking onset (Vetreno et al. 2013). Adolescent drinking is known to increase risk of developing alcohol dependence, with the risk decreasing with every year of delaying alcohol-use initiation across adolescence (for more information, see the sidebar). Studies found that in the OFC, a negative correlation existed between HMGB1–TLR4 expression and age at drinking onset, with lower HMGB1–TLR4 expression in individuals who initiated alcohol use later. The second correlation involved the amount of alcohol consumed, with total lifetime alcohol consumption positively correlated with OFC expression of HMGB1, TLR4, TLR3, TLR2, and RAGE (Crews et al. 2013). These findings further support the role of neuroimmune signaling in alcoholic brain and alcoholic neurodegeneration.

## Role of Microglia in Mediating Alcohol Actions in the Brain

Given their role in facilitating inflammation, it is not surprising that alcohol-activated microglia have been implicated in alcohol-induced inflammatory pathways. In rats, intermittent and chronic alcohol exposure can activate microglia while concomitantly increasing expression of proinflammatory cytokines and neuronal cell death, providing indirect evidence for the role of microglia in alcohol-induced neuroinflammation and neurotoxicity (Alfonso-Loeches and Guerri 2011; Chastain and Sarkar 2014; Zhao et al. 2013). Alcohol can activate microglia directly, via stimulation of TLRs, or indirectly, via neuronal damage and subsequent release of damage-associated molecular patterns that include HMGB1, resulting in the accumulation of microglia in the brain (i.e., reactive microgliosis) (Alfonso-Loeches and Guerri 2011). Microglial TLR4 seems to be necessary in alcohol-induced activation of microglia and subsequent microglial production of inflammatory mediators and apoptosis of neighboring neurons (Fernandez-Lizarbe et al. 2009, 2013). In an in vitro study (Boyadjieva and Sarkar 2010), microglia-conditioned media enhanced ethanol-induced apoptosis of cultured hypothalamic neurons. Interestingly, the neuronal cell death induced by microglia-conditioned media could be abolished if TNF-α was inactivated in the cultured cells, suggesting that microglial TNF-α production plays a key role in ethanol-induced neurotoxicity in developing neurons. The mechanism by which alcohol induces neuronal cell death may involve upregulation of NF-κB expression, which then stimulates release of TNF-α, resulting in neuronal apoptosis (Crews and Nixon 2009; Guadagno et al. 2013). Stimulation of the transcription factor AP-1 and release of IL-1β, IL-6, and transforming growth factor β (TGF-β1) also may contribute to alcohol-induced neuronal apoptosis (Alfonso-Loeches and Guerri 2011; Chen et al. 2006).

In addition to releasing cytokines, stimulated microglia contribute to neurotoxicity by secreting ROS (Takeuchi 2010). ROS, such as superoxide, hydrogen peroxide, and nitric oxide, can break down cell membranes and induce cell death. After alcohol exposure, ROS levels increase both as a natural byproduct of alcohol metabolism and as a result of enhanced cellular respiration, thus creating oxidative stress and leading to neuronal cell death (Guerri et al. 1994; Montoliu et al. 1995). Several studies have implicated microglia in the alcohol-induced production of ROS and resulting neurotoxicity. Qin and Crews (2012*a*) demonstrated that mice exposed to chronic alcohol showed increased levels of NADPH oxidase, superoxide, microglial activation, and cell death in cortical and hippocampal brain regions. Inhibition of NADPH oxidase during alcohol administration decreased superoxide, microglial activation, and cell death, directly linking ROS production to alcohol-induced microglial activation and neurotoxicity. In accord with these in vivo findings, in vitro studies showed that microglia-conditioned media enhanced ethanol-induced ROS production and oxidative stress in cultured hypothalamic neuronal cells and increased apoptotic cell death (Boyadjieva and Sarkar 2013*a*). Through these mechanisms, as well as the ethanol-related decreases in antioxidants discussed earlier, ethanol-activated microglia can induce apoptotic cell death and cell death in cultured fetal hypothalamic neurons from rat, suggesting that microglia may help facilitate ethanol-induced neurotoxicity by ROS.

Another cellular signaling mechanism by which alcohol induces neuronal apoptosis involves increased neuronal release of TGF-β1. Alcohol-induced elevation of TGF-β1 levels in neuronal cells is accompanied by a host of molecular and chemical changes related to cell death, including the following (Chen et al. 2006; Kuhn and Sarkar 2008):

- Increased expression of a protein called E2F1, whose overexpression sensitizes cells to apoptosis;
- Reduced expression of two key regulators of cell-cycle progression (i.e., cyclin D1 and cyclin-dependent kinase-4);
- Elevated levels of mitochondrial proapoptotic proteins bak, bad, and bcl-xs;
- Lowered levels of the antiapoptotic protein bcl-2; and
- Increased production of the apoptotic enzyme caspase 3.

Interestingly, in transformed cells, inhibition of NF-κB or ROS abrogates TGF-β1 stimulation of cell functions (Tobar et al. 2010). Hence, the ROS–NF-κB–TGF-β1 signaling cascade is a possible mechanism by which alcohol induces the apoptotic process in neurons—a process that is modulated by microglia. Another mechanism might relate to the microglial ability to reduce production of BDNF and cyclic adenosine monophosphate (cAMP) in neurons following ethanol activation. Thus, hypothalamic neuronal cell cultures treated with ethanol-activated microglia-conditioned medium showed decreased levels of both of these compounds. Treatment with BDNF or dibutyryl cAMP decreased the changes in the levels of intracellular free radicals, ROS, nitrite, glutathione, and catalase as well as neuronal apoptotic cell death that otherwise occurred when these cultures were treated with ethanol-activated microglia-conditioned medium. These findings suggest that ethanol increases the production of certain microglia-derived factors, thereby reducing cellular levels of cAMP and BDNF and increasing cellular oxidative stress and apoptosis in neuronal cells (Boyadjieva and Sarkar 2013*b*). However, further studies are needed to fully elucidate the mechanism(s) by which ethanol-activated signaling induces neuronal death.

Microglia also may mediate the effects of alcohol administration on the development of new neurons (i.e., neurogenesis). Alcohol exposure can result in decreased hippocampal neurogenesis, an effect that may underlie alcohol-related neurodegeneration (Crews et al. 2006; Morris et al. 2010). However, alcohol exposure followed by a period of abstinence results in increased hippocampal neurogenesis, which may serve a regenerative purpose. Interestingly, this process is preceded by microglial proliferation, raising the possibility that microglia may facilitate some regenerative mechanisms in recovery from alcohol exposure (McClain et al. 2011; Nixon et al. 2008).

In addition to being implicated in alcohol-induced neurotoxicity, microglia also might contribute to the processes that lead to the development of alcohol use disorder. Recent studies in rodents support a role for microglia in voluntary alcohol drinking and preference. In a quantitative-trait locus analysis of six strains of mice that differ in voluntary alcohol-drinking behavior, alcohol-preferring animals exhibited an increase in the expression of β-2-microglobulin, an NF-κB target gene involved in microglial MHC immune signaling (Mulligan et al. 2006). In addition, knockout of the β-2-microglobulin gene in mice decreased voluntary alcohol consumption and preference (Blednov et al. 2012). Finally, treatment with minocycline, an antibiotic and selective inhibitor of microglia, reduced voluntary alcohol consumption in adult mice (Agrawal et al. 2014). These studies suggest microglia might mediate alcohol preference and might contribute to the development of alcohol use disorder.

## Neuroimmune Signaling Integrates CNS Responses to Alcohol and Stress

### The Stress Axis and the Peripheral Immune System

Alcohol activation of immune signals and cytokine production in the brain affects not only cellular functions in the brain but also immune-system function in the periphery. The body’s main stress response systems—the hypothalamic–pituitary–adrenal (HPA) axis and the sympathetic nervous system (SNS)—are major pathways by which the brain and the immune system communicate. When the HPA axis is activated by a stressful situation, the hypothalamus releases corticotropin-releasing hormone (CRH), which acts on the pituitary to induce the release of adrenocorticotropic hormone. This hormone in turn acts on the adrenal glands to stimulate the release of stress hormones (i.e., glucocorticoids), including cortisol in humans and corticosterone in rodents. These hormones then help coordinate the body’s response to the stress. The SNS is part of the autonomic nervous system that regulates the body’s unconscious activities to maintain its normal functions. One of the main processes coordinated by the SNS is the fight-or-flight response to stress.

**Figure 6:**
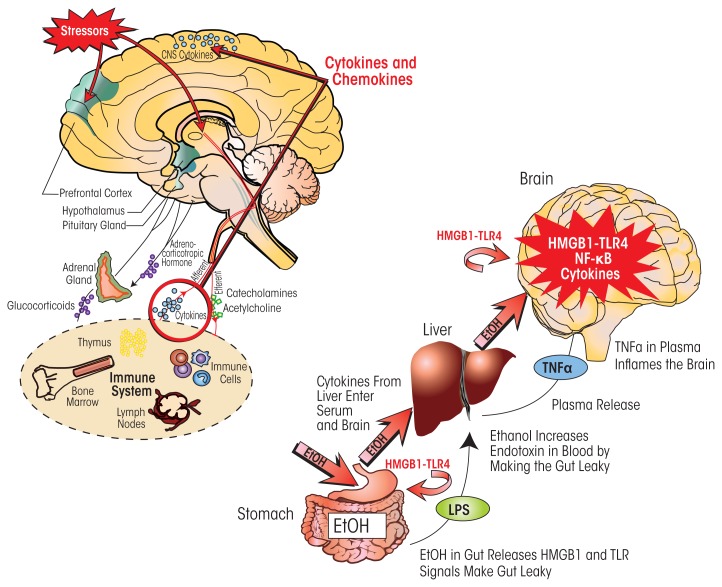
Neuroimmune signaling integrates central nervous system (CNS) responses to alcohol and stress. **(Left)** Stressors activate the body’s stress response system, which is comprised of the hypothalamus, pituitary gland, and adrenal glands (i.e., HPA axis) as well as the stress hormones they produce (e.g., adrenocorticotropic hormone and glucocorticoids). Stress also activates the sympathetic nervous system, which secretes catecholamines. These hormones act on various organs and tissues that are part of the immune system. In response, immune cells secrete cytokines that via the blood are transported to the brain. There, these cytokines lead to brain neuroimmune-gene induction that sensitizes stress-response pathways. At the same time, the immune system communicates with the CNS through sensory (afferent) nerves that activate the brain in response to stressful stimuli. This communication pathway involves particularly the vagus nerve and the nucleus tractus solitarius in the brain stem. **(Right)** Alcohol influences neuroimmune signaling via its effects on the gastrointestinal tract. Consumed ethanol enters the stomach and gut and makes them “leaky” by inducing the release of high-mobility group box 1 (HMGB1), which in turn activates Toll-like receptor 4 (TLR4) in the gut. As a result, bacterial products such as lipopolysaccharide (LPS) can enter the blood and reach the liver. Both LPS and ethanol (which also reaches the liver via the circulation) contribute to inflammatory reactions in the liver, which lead to release of tumor necrosis factor-alpha (TNF-α) and other proinflammatory cytokines from the liver. These proinflammatory cytokines in the blood enter the brain and increase neuroimmune-gene expression. Chronic ethanol also increases expression of HMGB1–TLR4 signaling in the brain, leading to persistent and progressive increases in neuroimmune-gene expression in the brain.

Alcohol has a potent activating effect on the HPA axis as well as on neuroimmune signaling; therefore, these effects may integrate the responses of the central nervous system (CNS) to alcohol and stress (figure 6). For example, TNF-α, IL-1β, and IL-6 act upon the HPA axis and SNS, both directly via local effects and indirectly via the CNS (Besedovsky and del Rey 1996; Pickering et al. 2005; Wilder 1995). Furthermore, CRH has a variety of complex effects on immune cells (Elenkov et al. 1999) and modulates immune/inflammatory responses through receptor-mediated actions of glucocorticoids on anti-inflammatory target immune cells (Tsigos and Chrousos 2002). In contrast, elevated glucocorticoid levels in the prefrontal cortex are proinflammatory, potentiating LPS–TLR4 activation of NF-κB and other proinflammatory signals (Munhoz et al. 2010). The neuro-transmitter norepinephrine that is released by SNS activation also disturbs inflammatory cytokine networks and innate immune-cell function. Similarly, the hypothalamic peptide β-endorphin (BEP), whose release is stimulated by CRH during HPA activation, can inhibit stress-hormone production and activate peripheral immune functions (Sarkar and Zhang 2013). All of these findings suggest that the stress–HPA axis, commonly thought to involve anti-inflammatory glucocorticoid actions, also contributes to stress–alcohol responses in the brain that can increase proinflammatory HMGB1–TLR–cytokine signaling.

HPA hormones influence the immune system in multiple ways (figure 6). Glucocorticoids prevent the migration of leukocytes from the circulation into extravascular regions, reduce accumulation of various immune cells (i.e., monocytes and granulocytes), and suppress the production and/or action of many cytokines and inflammatory mediators (Hermann et al. 1995; Sheridan et al. 1998; Zhang et al. 1998). They also inhibit a number of cytokines, including IL-1α, IL-1β, IL-6, IL-12, IFN-γ, TNF-α, granulocyte-macrophage colony-stimulating factor, and chemokine (C-C motif) ligand 5 (RANTES) (Sapolsky et al. 2000; Wiegers et al. 2005). At the same time, glucocorticoids increase the activity of TGF-β by activating a latent form of the cytokine (Oursler et al. 1993), which may indirectly affect the immune response, because TGF-β inhibits activation of T cells and macrophages. Glucocorticoids also increase production of IL-10, an anti-inflammatory cytokine that blocks NF-κB transcription and inhibits antigen presentation and T-cell activation (de Waal Malefyt et al. 1991). Finally, glucocorticoids suppress maturation, differentiation, and proliferation of immune cells, including innate immune cells, T cells, and B cells.

**Adolescence and Persistent Neuroimmune Expression in the Brain**

Adolescence is a developmental stage characterized by increased play behavior, thrill seeking, risk taking, puberty, and transition to independence. During this stage, the brain continues to develop; in particular, the frontal cortex continues to exhibit structural changes that coincide with maturation of adult behaviors and executive functions (Ernst et al. 2009). The developing brain is uniquely sensitive to alcohol, making adolescence a critical period of risk for developing alcohol use disorder (AUD) (Crews et al. 2007). Adolescence also is a period of experimentation, as exemplified by findings that alcohol-use initiation use typically begins during those years. The age of drinking onset is associated with various alcohol-related characteristics, including prevalence of lifetime AUD, as well as violence, fights, and injuries associated with alcohol use (Brown et al. 2008; Dawson et al. 2008; Sher and Gotham 1999). The younger the age of drinking onset, the more likely the person will develop AUD. In addition, binge drinking peaks during late adolescence.

The high prevalence of binge drinking among adolescents increases the importance of understanding how binge drinking might affect the adolescent brain. Studies found that a younger age of drinking onset is associated not only with an increased risk of lifetime AUD but also correlates with a smaller brain size and greater expression of high-mobility group box 1 (HMGB1) and Toll-like receptor 4 (TLR4), as well as other neuroimmune signaling receptors (Vetreno et al. 2013). These associations likely result both from pre-existing conditions that mature into dysfunctional behavior and from alcohol-induced factors than change over the life course and increase dysfunctional behavior, perhaps by altering brain maturation. The contributions of these two factors can only be determined by controlled experiments in which adolescent alcohol exposure is the only variable and genetic and other factors play no role. Such studies cannot be done in humans but are being done in animals (primarily rats) whose genetic background and environment can be controlled. The essential need to understand the neurobiology and impact of adolescent drinking on adulthood resulted in the formation of a consortium called NADIA, funded by the National Institute on Alcohol Abuse and Alcoholism, which addresses the contribution of adolescent alcohol abuse to adult psychopathology.

Adolescents have an immature response to alcohol, characterized by unique factors that differ from the adult response to alcohol. For example, adolescent rats show greater ethanol-induced memory impairment in certain tasks (e.g., the Morris water maze and discrimination tasks) than do adults (Land and Spear 2004; Markwiese et al. 1998). Similarly, humans who initiate alcohol use in their early 20s are more sensitive to the effects of alcohol on multiple memory tasks compared with those who start drinking in their late 20s (Acheson et al. 1998). Also, compared with adults, adolescents exhibit more potent inhibition of NMDA receptor-mediated synaptic activity in the hippocampus (Swartzwelder et al. 1995) as well as greater induction of long-term potentiation (LTP) (Martin et al. 1995). Adolescents, who already exhibit social behaviors, also are uniquely sensitive to the social facilitative effects of ethanol (Varlinskaya and Spear 2002). Consistent with findings in humans, adolescent rats are more sensitive to binge-drinking models of brain damage, particularly in the frontal cortex (Crews et al. 2006). Interestingly, adolescent rats are less sensitive than adults to certain effects of alcohol, such as the sedative (Little et al. 1996; Silveri and Spear 1998), motor impairing (Little et al. 1996, White et al. 2002*a*,*b*), social inhibitory (Varlinskaya and Spear 2002), and aversive (Anderson et al. 2010) effects. Adolescent rats also show electrophysiological differences from adults in the hippocampus, particularly a reduced sensitivity to γ-aminobutyric acid (GABA) type A (GABAA) receptor-mediated inhibition (Carr et al. 2003; Sullivan et al. 2006; Yan et al. 2009, 2010). The reduced sedative sensitivity to alcohol and increased alcohol-induced cognitive disruption observed in adolescent animals is consistent with findings in humans that adolescents have high rates of binge drinking and are at particularly high risk of alcohol-related traffic crashes. The continuous increase in high binge-drinking levels in human adolescents over the past decade justifies the need to study the long-term consequences of adolescent alcohol abuse in more detail.

Like adult alcohol exposure, adolescent exposure induces neuroimmune genes in the brain; furthermore, in humans, the effect on neuroimmune genes correlates with age of drinking onset. Indeed, Vetreno and Crews (2012) found that intermittent binge-ethanol treatment in adolescent rats increased expression of multiple innate immune genes in the frontal cortex during adulthood. Interestingly, whereas expression of the critical neuroimmune signaling receptor TLR4 decreased during adolescence in controls, expression of this receptor increased and remained elevated into adulthood in adolescents with binge ethanol exposure (Vetreno and Crews 2012). In contrast, the expression of HMGB1 in the frontal cortex increased during adolescence in control subjects, and this increase was exacerbated by adolescent binge ethanol exposure. Moreover, adolescent alcohol exposure resulted in a persistent increase in adult HMGB1 and TLR4 levels that may represent adolescent-like HMGB1–TLR4 signaling in these adults.

As mentioned in the main article, HMGB1–TLR4 signaling induced by alcohol exposure can enhance sensitivity at the NMDA glutamate receptor, which can counteract ethanol’s direct inhibitory effects on this receptor. Accordingly, adults with persistent increases in HMGB1–TLR4 signaling resulting from adolescent alcohol exposure might experience adolescent-like tolerance to alcohol’s sedative effects, and perhaps increased adolescent-like cognitive disruption as well. Although adolescent alcohol exposure does not markedly disrupt adult learning tasks, adolescent intermittent binge exposure induces deficits in reversal learning in adult rats (Vetreno and Crews 2012) and mice (Coleman et al. 2011). These studies are consistent with the hypothesis that the adolescent brain is vulnerable to long-lasting changes that persist through maturation into adulthood. Persistent neuroimmune-gene induction likely contributes to continuous slow neurodegeneration as well as to more specific insults on key neurotransmitters that mature during adolescence (Crews et al. 2007; Vetreno and Crews 2012) and may also be related to a persistent loss of behavioral flexibility. Together, the persistent loss of ability to adapt to changes, low sedative response to alcohol, and increased sensitivity to cognitive disruption associated with adolescent alcohol exposure all are likely to promote and sustain high alcohol-drinking levels. These in turn will promote more alcohol consumption and the chances that AUD will develop in addition to alcoholic neurodegeneration.

—*Fulton T. Crews, Ph.D.; Dipak K. Sarkar, Ph.D., D.Phil.; Liya Qin, Ph.D.; Jian Zou, Ph.D.; Nadka Boyadjieva, M.D., Ph.D., D.Sci.; Ryan P. Vetreno, Ph.D.*

ReferencesAchesonSKSteinRMSwartzwelderHSImpairment of semantic and figural memory by acute ethanol: Age-dependent effectsAlcoholism: Clinical and Experimental Research227143714421998980252510.1111/j.1530-0277.1998.tb03932.xAndersonRIVarlinskayaEISpearLPEthanol-induced conditioned taste aversion in male Sprague-Dawley rats: Impact of age and stressAlcoholism: Clinical and Experimental Research34122106211520102086061810.1111/j.1530-0277.2010.01307.xPMC2988942BrownSAMcGueMMaggsJA developmental perspective on alcohol and youths 16 to 20 years of agePediatrics121Suppl 4S290S31020081838149510.1542/peds.2007-2243DPMC2765460CarrLGSpenceJPPeter ErikssonCJAA and ANA rats exhibit the R100Q mutation in the GABAA receptor alpha 6 subunitAlcohol311–29310720031461501610.1016/j.alcohol.2003.07.003ColemanLGJrHeJLeeJAdolescent binge drinking alters adult brain neurotransmitter gene expression, behavior, brain regional volumes, and neurochemistry in miceAlcoholism: Clinical and Experimental Research35467168820112122330410.1111/j.1530-0277.2010.01385.xPMC3544413CrewsFHeJHodgeCAdolescent cortical development: A critical period of vulnerability for addictionPharmacology, Biochemistry, and Behavior86218919920071722289510.1016/j.pbb.2006.12.001PMC11646682CrewsFTMdzinarishviliAKimDNeurogenesis in adolescent brain is potently inhibited by ethanolNeuroscience137243744520061628989010.1016/j.neuroscience.2005.08.090DawsonDAStinsonFSChouSPGrantBFThree-year changes in adult risk drinking behavior in relation to the course of alcohol-use disordersJournal of Studies on Alcohol and Drugs69686687720081892534510.15288/jsad.2008.69.866PMC2583373ErnstMRomeoRDAndersenSLNeurobiology of the development of motivated behaviors in adolescence: A window into a neural systems modelPharmacology, Biochemistry, and Behavior93319921120091913602410.1016/j.pbb.2008.12.013LandCSpearNEFear conditioning is impaired in adult rats by ethanol doses that do not affect periadolescentsInternational Journal of Developmental Neuroscience225–635536220041538083510.1016/j.ijdevneu.2004.04.008LittlePJKuhnCMWilsonWASwartzwelderHSDifferential effects of ethanol in adolescent and adult ratsAlcoholism: Clinical and Experimental Research208134613511996894730910.1111/j.1530-0277.1996.tb01133.xMarkwieseBJAchesonSKLevinEDDifferential effects of ethanol on memory in adolescent and adult ratsAlcoholism: Clinical and Experimental Research22241642119989581648MartinDTayyebMISwartzwelderHSEthanol inhibition of AMPA and kainate receptor-mediated depolarizations of hippocampal area CA1Alcoholism: Clinical and Experimental Research195131213161995856130710.1111/j.1530-0277.1995.tb01617.xSherKJGothamHJPathological alcohol involvement: A developmental disorder of young adulthoodDevelopment and Psychopathology11493395619991062473310.1017/s0954579499002394SiveriMMSpearLPDecreased sensitivity to the hypnotic effects of ethanol early in ontogenyAlcoholism: Clinical and Experimental Research2236706761998962244910.1111/j.1530-0277.1998.tb04310.xSullivanEVAdalsteinssonESoodRLongitudinal brain magnetic resonance imaging study of the alcohol-preferring rat. Part I: Adult brain growthAlcoholism: Clinical and Experimental Research3071234124720061679257210.1111/j.1530-0277.2006.00145.xSwartzwelderHSWilsonWATayyebMIDifferential sensitivity of NMDA receptor-mediated synaptic potentials to ethanol in immature versus mature hippocampusAlcoholism: Clinical and Experimental Research1923203231995762556410.1111/j.1530-0277.1995.tb01509.xVarlinskayaEISpearLPAcute effects of ethanol on social behavior of adolescent and adult rats: Role of familiarity of the test situationAlcoholism: Clinical and Experimental Research26101502151120021239428310.1097/01.ALC.0000034033.95701.E3VetrenoRPCrewsFTAdolescent binge drinking increases expression of the danger signal receptor agonist HMGB1 and Toll-like receptors in the adult prefrontal cortexNeuroscience22647548820122298616710.1016/j.neuroscience.2012.08.046PMC3740555VetrenoRPQinLCrewsFTIncreased receptor for advanced glycation end product expression in the human alcoholic prefrontal cortex is linked to adolescent drinkingNeurobiology of Disease59526220132386723710.1016/j.nbd.2013.07.002PMC3775891WhiteAMBaeJGTruesdaleMCChronic-intermittent ethanol exposure during adolescence prevents normal developmental changes in sensitivity to ethanol-induced motor impairmentsAlcoholism: Clinical and Experimental Research2679609682002a1217010410.1097/01.ALC.0000021334.47130.F9WhiteAMGhiaAJLevinEDSwartzwelderHSBinge pattern ethanol exposure in adolescent and adult rats: Differential impact on subsequent responsiveness to ethanolAlcoholism: Clinical and Experimental Research24812511256200010968665WhiteAMTruesdaleMCBaeJGDifferential effects of ethanol on motor coordination in adolescent and adult ratsPharmacology, Biochemistry, and Behavior7336736772002b1215104310.1016/s0091-3057(02)00860-2YanHLiQFlemingRDevelopmental sensitivity of hippocampal interneurons to ethanol: Involvement of the hyperpolarization-activated current, IhJournal of Neurophysiology1011678320091897129810.1152/jn.90557.2008PMC2637011YanHLiQMadisonRDifferential sensitivity of hippocampal interneurons to ethanol in adolescent and adult ratsJournal of Pharmacology and Experimental Therapeutics3351516020102066012610.1124/jpet.110.168450PMC2957785

Norepinephrine released after SNS activation also disturbs inflammatory cytokine networks by inhibiting production of immune-enhancing cytokines, such as IL-12 and TNF-α, and by upregulating production of inhibitory cytokines, such as IL-10 and TGF-β (Webster et al. 2002). Additionally, norepinephrine affects peripheral natural killer (NK) cells, a subset of lymphocytes that are a first-line defense against viral infections, tumor growth, and metastasis via their unique cytolytic action (Herberman and Ortaldo 1981). These cells carry receptors for norepinephrine (i.e., β-adrenergic receptors) on their surfaces (Madden et al. 1995). The cytolytic activity of NK cells involves the synergistic actions of the pore-forming protein perforin and the serine protease granzyme B to cause apoptosis of target cells (Graubert et al. 1996). Among the HPA hormones, gluco-corticoids and CRH both are potent inhibitors of NK-cell activity in vitro and in vivo. Hypothalamic CRH inhibits NK activity and IFN-γ production through actiavation of the SNS, which causes release of catecholamines (e.g., norepinephrine) from the spleen and activation of β-adrenergic receptors on NK cells (Irwin et al. 1990). Thus, it appears that the hormones secreted during stress by the HPA axis and SNS have inhibitory effects on peripheral immune functions that contrast with their actions in the CNS.

During activation of the HPA axis, secretion of CRH and catecholamines also increases the secretion of BEP in the hypothalamus. In a feedback mechanism, BEP regulates the secretion of CRH in a hypothalamic region called the paraventricular nucleus (PVN) (Plotsky 1986). In the PVN, the BEP-releasing neurons act on CRH-releasing neurons and inhibit CRH release, thus regulating the activity of the stress system (Plotsky et al. 1993). BEP acts by binding to δ- and μ-opioid receptors; accordingly, treatment with a μ-opioid receptor antagonist results in increased CRH release (Boyadjieva et al. 2006). BEP affects immune-system function through a variety of mechanisms. By binding to δ- and μ-opioid receptors BEP modulates neurotransmission in sympathetic and parasympathetic neurons via neuronal circuitry within the PVN, ultimately resulting in activation of NK-cell cytolytic functions in the spleen (Boyadjieva et al. 2006, 2009; Sarkar et al. 2011). If incubated with human bone marrow mononuclear cells or NK-enriched cell populations, BEP enhances NK activity (Mathews et al. 1983). In animal models, chronic BEP infusion into the blood vessels in the brain enhances NK-cell activity in vivo, and this effect is eliminated by the opioid antagonist naloxone (Jonsdottir et al. 1996). BEP also can inhibit T-cell proliferation (van den Bergh et al. 1993) as well as antibody production (Morgan et al. 1990).

Abnormalities in BEP neuronal function are correlated with a higher incidence of cancers and infections in patients with schizophrenia, depression, and fetal alcohol syndrome and in obese patients (Bernstein et al. 2002; Lissoni et al. 1987; Polanco et al. 2010; Zangen et al. 2002). Interestingly, BEP-cell transplantation in the hypothalamus suppresses various cancers in rat models by activating innate immune-cell functions and altering inflammatory and anti-inflammatory cytokine milieus (Sarkar et al. 2008). In this setting, chronic alcohol use suppresses BEP neuronal activity and is connected with increased infection rates and higher incidence of cancer. Thus, alcohol and stress seem to paralyze adaptive innate immune functions by inducing complex changes in NK cells and other adaptive immune signaling that in the brain primarily involves microglial–astrocyte–neuronal HMGB1–TLR signaling.

## Effects of Immune System Activation on Brain Function

The interaction between the brain and the immune system is not unidirectional—that is, immune-system responses also may influence responses in the brain. Recent studies indicate that ethanol causes HMGB1 release in the gut, which activates TLR4. As a result, the gut leaks LPS-like bacterial products, thereby stimulating proinflammatory cytokine induction in the liver, which in turn leads to increased levels of TNF-α and other cytokines in the blood. Qin and Crews (2007, 2012*b*) discovered that LPS-induced increases in serum TNF-α as well as proinflammatory cytokines led to gene induction in the brain. The proinflammatory cytokines in the blood can be transported across the blood–brain barrier (BBB) by their receptors (e.g., TNFR) (Banks and Erickson 2010; Qin et al. 2007). Using intraperitoneal injections of LPS to stimulate proinflammatory responses in the liver and other tissues and induce proinflammatory cytokines, researchers discovered parallel increases in TNF-α in the blood and brain (Qin et al. 2007). In transgenic mice lacking TNF receptors, however, LPS increased TNF-α only in the blood but not in the brain, suggesting that LPS–TLR4 induction of TNF-α in the blood leads to TNF transport by its receptors across the BBB and activation of proinflammatory responses in the brain. Transgenic mice without the TNF-α receptor cannot transport the cytokine to the brain; consequently, the LPS–TLR4 proinflammatory response is amplified across peripheral tissues but does not spread to the brain.

Ethanol can increase proinflammatory cytokine levels in the blood by activating proinflammatory responses in the liver and other tissues. One mechanism seems to involve the ethanol-induced increase in gut permeability (or “leakiness”) mentioned above (Ferrier et al. 2006). At high doses (at least 2 to 3 g/kg ethanol administered into the stomach), ethanol potentiates innate immune signaling in the gut (Ferrier et al. 2006). This disrupts the connections between the cells lining the gut (i.e., gut tight junctions) and opens sites that allow gut bacteria and their endotoxins to enter the blood vessels leading to the liver, where they can initiate a proinflammatory response (Sims et al. 2010). Thus, high doses of ethanol increase systemic proinflammatory responses, which can then spread to the brain through TNF-α and likely other cytokines (see figure 6).

Although some in vitro studies have suggested that ethanol can interfere with the BBB, most in vivo studies do not show BBB damage following chronic ethanol treatment. Marshall and colleagues (2013) assessed BBB integrity by tracking a protein (i.e., albumin) that cannot cross an intact BBB in rats that were administered large amounts of alcohol for 4 days (a regimen that can induce alcoholic brain damage). The analyses found no evidence of albumin in the brain, indicating that the BBB had remained intact following the ethanol treatment. Using the same model, Crews and colleagues (2006) found that inhibition of NF-κB protected against the brain damage and inhibition of neurogenesis normally induced by this regimen. These findings are consistent with the assumption that proinflammatory responses in the brain mediate brain damage without causing BBB damage. Instead, the brain damage may be induced through direct activation of proinflammatory responses in the brain and/or systemic proinflammatory signals that are transported across the BBB and contribute to brain proinflammatory responses.

**Glossary**

Antibody: Immune molecule (protein) produced by B cells that recognizes foreign molecules that have entered the body (i.e., antigens), binds to these molecules, and marks them for destruction by the body’s immune system
Astrocytes: Characteristic star-shaped non-neuronal cells in the brain and spinal cord that support the endothelial cells that form the blood–brain barrier and provide nutrients to the nervous tissue
Cytokine: Any of a group of molecules, produced primarily by immune cells, that regulate cellular interactions and other functions; many cytokines play important roles in initiating and regulating inflammatory reactions
Endotoxin: A highly toxic chemical component of the cell walls of certain bacteria that occur normally in the intestine. Endotoxin can be released into the bloodstream when bacteria die or there is an increase in gut permeability
Excitotoxicity: Pathological process by which nerve cells are damaged or killed after being excessively stimulated by excitatory neurotransmitters (e.g., glutamate)
Kinase: An enzyme that transfers phosphate groups from one molecule (the donor) to a specific target molecule (the substrate)
Long-term potentiation (LTP): Process by which an episode of strong receptor activation at a synapse leads to a subsequent long-lasting strengthening of the signal transmission across that synapse (e.g., by inducing the accumulation of more receptor molecules at that synapse)
Macrophages: A type of immune cell that ingests foreign particles and microorganisms in a process called phagocytosis and which synthesizes *cytokines* and other molecules involved in inflammatory reactions
Major histocompatibility complex (MHC): A highly diverse set of glycoproteins in the cell membranes of almost all cells that help to present foreign molecules (i.e., antigens) to other immune cells (i.e., T cells) to activate these cells and induce an immune response
Microglia: Type of non-neuronal cell in the central nervous system (CNS) that acts as the first and main form of active immune defense in the CNS
Monocytes: A type of white blood cell involved in the innate immune response; upon activation (e.g., in response to an infection) they move to the site of the infection, enter the tissues, and differentiate into *macrophages*, which then can engulf and destroy the pathogen
Sepsis: The presence of pathogenic organisms or their toxic products in the blood or tissues
Toll-like receptors (TLRs): A class of proteins that play a key role in innate immunity. They are located on *macrophages* as well as other brain cells (i.e., *astrocytes* and neurons) and are activated in response to various pathogens; this activation triggers additional innate immune responses and, eventually, adaptive immune responses

Although the levels of proinflammatory gene expression in the blood and brain parallel each other at early time points after initiation of an immune response, the brain’s response to LPS is much smaller than that found in the liver and blood during the first few hours. Surprisingly, the blood and liver responses to LPS return to baseline over about 8 to 12 hours, whereas the increase in proinflammatory gene expression in the brain persists for months. This leads to degeneration of dopamine neurons in the substantia nigra, a region in the midbrain involved in reward and addiction (Qin et al. 2007). Similarly, liver and blood responses to binge alcohol exposure appear to be small and transient, although they have not been extensively investigated. In contrast, brain expression of the proinflammatory cytokine MCP-1 persists for at least 1 week (Qin et al. 2008).

Exposure of C57BL/6 mice to 10 daily doses of ethanol followed by LPS results in increased LPS induction of proinflammatory cytokines in the liver, blood, and brain compared with control animals treated only with LPS (Qin et al. 2008). However, this ethanol-induced sensitization to the LPS response resulted in sustained increases in multiple proinflammatory cytokines, including TNF-α, IL-1β, and MCP-1 only in the brain, but not in the liver. The mechanism underlying the sustained brain response and transient liver response is not clear. The investigators noted that the anti-inflammatory cytokine IL-10, which inhibits NF-κB, was increased in the liver 1 week after alcohol treatment, but decreased in the brain (Qin et al. 2008). This suggests that anti-inflammatory mechanisms may contribute to the loss of the liver response. Further analyses found that mice pretreated with ethanol are sensitized not only to the TLR4 receptor agonist LPS but also to the TLR3 agonist Poly:IC (Qin and Crews 2012*a*). Similar to LPS, Poly:IC induces proinflammatory genes in the brain at 24 hours after 10 days of daily alcohol administration (5 g/kg/day). These findings suggest that chronic ethanol sensitizes proinflammatory TLR responses that are easily observed after the clearance of alcohol.

Taken together, the observations indicate that chronic ethanol sensitizes both systemic and brain responses to neuroimmune-gene activation through induction of HMGB1 and TLR proteins. Ethanol-induced leaky gut occurs after high binge-drinking doses, with gut ethanol exposure often being equivalent to the beverage content (i.e., 80 proof is 40 percent ethanol). As a result, bacterial products enter the circulation to the liver and activate liver monocytes (i.e., Kupffer cells), which then produce cytokines, including TNF-α. The TNF-α can be transported to the brain, activating brain neuroimmune signaling that persists for long periods (Qin et al. 2007). Thus, at least two mechanisms of ethanol activation of neuroimmune signaling exist—a direct activation within the brain and the spread of a systemic innate immune activation to the brain (figure 6).

## Summary

Binge drinking stimulates neuroimmune-gene induction, which increases neurodegeneration through increased oxidative stress, particularly NADPH oxidase-induced oxidative stress. In addition, HMGB1–TLR4 and NF-κB signaling are increased, leading to enhanced expression of NF-κB target genes and, ultimately, to persistent and sensitized neuroimmune responses to ethanol and other agents that release HMGB1 or directly stimulate TLR receptors and/or NMDA receptors. Persistent neuroimmune-gene induction alters stress-coping mechanisms and the sympathetic nervous system, resulting in the HPA-mediated enhancement of peripheral cytokines, which further exacerbates the neuroimmune response. In addition to neuroimmune signaling, glutamate excitotoxicity also is linked to alcoholic neurodegeneration.

It has been proposed that, instead of simply being a side effect of excessive alcohol consumption, neuronal damage associated with drinking actually may underlie some of the mechanisms of developing alcohol use disorder (Crews and Boettiger 2009). The development of dependence is thought to result at least in part from a lack of inhibition of the subcortical mesolimbic reward system by the frontal cortex (Koob and Le Moal 1997). Alcohol-induced cell death in regions such as the prefrontal cortex may lead to lack of inhibition in subcortical reward areas such as the striatum, which in turn may reduce behavioral inhibition and increase motivation to drink. Repeated stimulation of the innate immune system during chronic or heavy alcohol consumption may facilitate this process, leading to decreased inhibition of the mesolimbic reward system and thus increased drinking (Crews et al. 2011). These processes may be particularly relevant in adolescence, when persistent and long-lasting increases in brain neuroimmune-gene expression and neurodegeneration may be associated with the development of alcohol use disorder.

## Footnotes

1 NADPH oxidase is an enzyme that produces reactive oxygen species (ROS)—for example, during ethanol metabolism—thereby increasing oxidative stress and contributing to cell damage.

2 HMGB1 also is known as amphoterin (Huttunen and Rauvala 2004).

3 Researchers have identified 13 TLRs (i.e., TLR 1-13) in mammals (Medzhitov 2001; Takeda et al. 2003); however, only TLR2, TLR3, and TLR4 have been assessed in alcoholic brain.
